# Impact of COVID-19 on the number of transplants performed in Brazil during the pandemic. Current situation

**DOI:** 10.1590/0100-6991e-20213042

**Published:** 2021-09-14

**Authors:** MARCELO AUGUSTO FONTENELLE RIBEIRO, CASSIA TIENI KAWASE COSTA, PAOLA REZENDE NÉDER, ISABELLA DE ALMEIDA AVEIRO, YASMIN GARCIA BATISTA ELIAS, SAMARA DE SOUZA AUGUSTO

**Affiliations:** 1 - Pontifícia Universidade Católica de São Paulo - PUCSP-Sorocaba, Disciplina de Cirurgia Geral e Trauma - Sorocaba - SP - Brasil; 2 - Faculdade de Ciências Médicas de São José dos Campos - Humanitas - São José dos Campos - SP - Brasil; 3 - Universidade de Santo Amaro, Curso de Medicina - São Paulo - SP - Brasil

**Keywords:** Coronavirus Infections, Transplants, Organ Transplantation, Tissue Donors, Living Donors, Infecções por Coronavirus, Transplante de Órgãos, Doadores de Tecidos, Transplantes, Doadores Vivos

## Abstract

The intense use of resources to combat COVID-19 causes concern in the entire transplant community because, in addition to physical limitations such as ICU beds, lack of homogeneous treatment protocols and uncertainties about the effects of immunosuppression on viral progression have significant impact on transplant surgeries. The aim of the present study is to comparatively assess the number of solid organ transplants performed in 2019 and 2020, as well as the impact of the COVID-19 pandemic on organ donation and transplant surgeries in Brazil. The last 10 years have shown increasing trend in the number of solid organ transplants, which have significantly decreased in 2020. Lung transplantations were mostly affected by the pandemic; these surgeries have been carried out only in Rio Grande do Sul and São Paulo states. Liver transplantations were the least affected ones, since the number of surgeries have only decreased by 10.8% in the first three quarters of 2020, in comparison to 2019. The number of active patients on the waiting list for heart and kidney transplantation has increased in 2020. Therefore, it is necessary developing strategies to keep the structure necessary for organ transplantation processes active and, consequently, to reduce the impacts of the pandemic on these patients.

## RESUMO

A utilização intensa dos recursos para o combate da COVID-19 causa preocupação em toda comunidade de transplantes, pois além das limitações físicas, como leitos de UTI, a falta de protocolos homogêneos para tratamentos e as incertezas dos efeitos da imunossupressão na progressão do vírus, resultam em um impacto significativo nas cirurgias de transplantes. O objetivo do presente estudo é avaliar comparativamente o número de transplantes de órgãos sólidos realizados nos anos de 2019 e 2020, e o impacto da pandemia na doação e transplantes de órgãos no Brasil. Considerando os últimos 10 anos, é possível observar uma tendência de aumento no número de transplantes de órgãos sólidos, com queda expressiva no ano de 2020. O transplante pulmonar foi o mais atingido pela pandemia, sendo realizado apenas nos estados do Rio Grande do Sul e São Paulo. O transplante hepático foi o menos afetado, apresentando uma diminuição de apenas 10,8% nos três primeiros trimestres de 2020, quando comparados com 2019. Sobre os pacientes ativos em lista de espera, houve um aumento em 2020 para transplante de coração e rim. Portanto, estratégias devem ser desenvolvidas para que a estrutura necessária ao processo de transplantes de órgãos se mantenha ativa, reduzindo assim os impactos da pandemia sobre estes pacientes.

## INTRODUCTION

The Coronaviridae family is globally known and presents high mutation ability^1^. The first COVID outbreak in humans was identified in China, in 2002; it is a zoonosis and was caused by Sars-CoV-I. A decade later, a new outbreak caused by the MERS-CoV (Middle East Respiratory Syndrome) emerged in Saudi Arabia. Most recently, in 2019, a series of pneumonia cases was identified in Wuhan City, Hubei province - China; the causative agent was identified as a single-stranded β-coronavirus, subsequently categorized as Sars-CoV-2 (Acute Respiratory Distress Syndrome), which presents higher infectivity than the previous virus^2^ ^,^ ^3^. 

The virus relates to the human body through the S protein (spike protein), which binds to the angiotensin-converting enzyme 2 (ACE2). The latter is widely expressed in human tissues, mainly those of the lower respiratory tract (in particular, in type II pneumocytes)^4^. Disease signs and symptoms - such as fever, tiredness/fatigue, dry cough, lack of appetite/anorexia, muscle pain, dyspnea, sore throat, runny nose, headache, nausea and diarrhea - tend to appear after the incubation time, which ranges from two to 14 days (five days, on average). Disease progression to respiratory failure is likely to happen in extreme cases, mainly in elderly individuals and/or in patients who have some comorbidity. It is essential to provide support to these patients in intensive care units (ICUs), where mechanical ventilation can be used as a life support resource^2^ ^,^ ^3^. 

The complexity of cases has worldwide overwhelmed the public health systems^5^. Intense resource use to fight the Covid-19 pandemic has raised concerns in the entire transplant community because in addition to the physical limitations such as ICU beds, the lack of homogeneous treatment protocols and uncertainties about the effects of immunosuppression on viral progression have significant impact on transplant operations^6^ ^,^ ^7^. Brazil is a world transplant reference, which is substantially performed and financed by the Brazilian Unified Health System (SUS - Sistema Único de Saúde)^8^. In total, 119,120 solid organ transplants were performed in the national territory in ten years (from 2009 to 2019). Kidney ranks first, with the absolute number of 6,283 transplant operations/year; it is followed by liver (2,245 transplant procedures/year), heart (378 transplant operations/year), pancreas (173 transplants/year) and lung (104 operations/year)^9^. 

The current pandemic has significant impact on transplant procedures^7^ ^,^ ^10^. A study carried out in the Netherlands has shown a decrease in the number of transplants by 67% in the first month of the pandemic, which, for example, increased the mortality rate among patients waiting for kidney transplantation^11^.

The aim of the present study was to comparatively assess the number of transplant operations performed in 2019 and 2020, as well as the impact of the COVID-19 pandemic on organ donation, in Brazil.

## METHOD

Data referring to the timeframe between January and September in 2019 and 2020 were collected in the Brazilian Transplantation Registry (RBT - Registro Brasileiro de Transplantes), which is the official information tool used by the Brazilian Organ Transplant Association (ABTO - Associação Brasileira de Transplante de Órgãos). This paper underwent the evaluation of the Research Ethics Committee of Hospital Moriah - São Paulo, registration number 003/2021.

## RESULT

Transplant operations included in the current study comprised solid organs such as heart, lung, liver, pancreas and kidney, from January to September 2019 and 2020^9^ ^,^ ^12^ (Table 1). 

**Table 1:** Number of solid organ transplants in Brazil, from January to September 2019 and 2020.

Organ	January to September 2019	January to September 2020
Heart	285	218
Lung	72	39
Liver	1,620	1,506
Pancreas	125	108
Kidney	4,617	3,486

The state analysis has shown that the number of potential and effective donors in the country ranged from 8,469 potential donors and 2,775 effective donors in 2019 to 7,725 potential donors and 2,438 effective donors in 2020^9^ ^,^ ^12^ (Table 2). In 2020, there was a decrease in the number of donors whose organs were transplanted^9^ ^,^ ^12^ (Table 3).

**Table 2:** Potential donors and effective donors by State, from January to September 2019 and 2020.

State	January to September 2019		January to September 2020	
	Potential donors	Effective donors	Potential donors	Effective donors
São Paulo (SP)	2,325	810	2,245	810
Paraná (PR)	836	352	855	361
Rio de Janeiro (RJ)	664	221	670	211
Minas Gerais (MG)	584	218	552	194
Santa Catarina (SC)	445	236	455	208
Rio Grande do Sul (RS)	512	182	422	140
Bahia (BA)	425	122	349	96
Ceará (CE)	442	183	336	123
Pernambuco (PE)	360	143	259	75
Goiás (GO)	337	58	243	54
Distrito Federal (DF)	221	39	242	30
Mato Grosso do Sul (MS)	178	38	156	35
Espírito Santo (ES)	163	33	152	29
Rio Grande do Norte (RN)	158	39	136	13
Paraíba (PB)	106	11	111	16
Sergipe (SE)	110	12	99	7
Maranhão (MA)	80	7	92	4
Amazonas (AM)	86	11	74	13
Rondônia (RO)	67	21	58	7
Mato Grosso (MT)	64	3	48	1
Pará (PA)	86	17	46	4
Piauí (PI)	86	4	39	4
Alagoas (AL)	63	13	33	1
Acre (AC)	37	3	28	0
Roraima (RR)	16	1	14	1
Tocantins (TO)	14	1	11	1
Amapá (AP)	4	0	0	0
Brazil	8.469	2.775	7.725	2.438

**Table 3:** Potential donors, effective donors and donors whose organs have been transplanted, from January to September 2019 and 2020.

January to September 2019			January to September 2020		
Potential donors	Effective donors	Donors whose organs were transplanted	Potential donors	Effective donors	Donors whose organs were transplanted
8.469	2.775	2.366	7.725	2.438	2.036

There was an increasing trend in the number of transplants of most solid organs in the last 10 years; however, this number was significantly decreased in 2020 (Table 4). This phenomenon can be easily visualized in Graphic 1, which was plotted by ABTO, based on annual projection for 2020^9^ ^,^ ^12^. 

**Table 4:** Number of solid organ transplants in Brazil, from 2010 to September 2020.

Organ	2010	2011	2012	2013	2014	2015	2016	2017	2018	2019	2020
Heart	166	160	228	272	311	353	357	380	357	378	291
Lung	61	49	69	80	67	74	92	112	121	104	52
Liver	1,412	1,497	1,603	1,726	1,758	1,810	1,882	2,122	2,195	2,256	2,007
Pancreas	133	181	153	143	128	121	135	113	146	175	144
Rim	4,654	4,982	5,431	5,465	5,661	5,591	5,531	5,930	5,949	6,295	4,646

Regarding donor characteristics, ABTO considers sex, age group, blood group and cause of death. It was observed a significant decrease in the number of donors due to traumatic brain injury (TBI) in 2020, although lower than in 2019^9^ ^,^ ^12^ (Table 5). 

**Table 5:** Profile of organ donors, 2019 and 2020.

		2019			2020	
	Traumatic brain injury	Brain stroke	Others	Traumatic brain injury	Brain stroke	Others
Total	1,034	1,903	480	556	900	282
Percentage	30%	56%	14%	32%	52%	16%

There was an increase in the number of active patients on the waiting list for heart and kidney transplantation in 2020^9^ ^,^ ^12^ (Table 6).

**Table 6:** Number of patients on the waiting list, by organ, until September 2019 and 2020.

Organ	September 2019	September 2020
Heart	260	277
Lung	199	195
Liver	1,124	1,100
Pancreas	24	18
Kidney	23,630	25,724

## DISCUSSION

The number of organ donors during the COVID-19 pandemic has decreased in Brazil, mainly the number of donors who died due to traumatic brain injury. This factor may be associated with the decreased number of individuals on the streets - at least in the first months of the pandemic - and, consequently, with a decreased number of accidents and trauma types^13^. The number of stroke-associated donors remained stable, as expected. Given such consequences and other reflexes of the pandemic, the number of transplant operations in the country decreased and that of patients on waiting lists increased. This new reality reduced the likelihood of patients to receive organs in shorter periods-of-time^9,12.^

### Heart

The heart transplant rate decreased by 23% in 2020, in comparison to that of 2019; the decrease of 49% observed in the 2^nd^ quarter of 2020 dropped to 13.4% in the 3^rd^ quarter. A significant drop was observed in the 2^nd^ and 3^rd^ quarters of 2020, in the Northeastern (8.9% and 47.6%) and Southern (64.7% and 57.1%) regions. The Southeast region, whose heart transplant rate decreased by 40.4% in the 2^nd^ quarter of 2020, increased by 2.1% in the 3^rd^ quarter, whereas the Midwestern region increased heart transplant rate in the latter two quarters (33.3% and 66.7%). The Federal District (8.0 pmp) stood out for liver and heart transplantation in 2020^9^. 

A French study analyzed data recorded during the pandemic from March to July 2020 and observed 1,530 transplant operations, in total - 167 of them were heart transplantations. There was an increase transplant activity after the program reopened, although there was a decrease in comparison to 2019^14^.

The volume of transplants from deceased donors in the USA significantly decreased in all the regions of the country in March and April 2020 - heart transplantations decreased by 43%. However, there was in the subsequent months, an increase was seen in the number of transplants due to efficient and safe actions taken by the Organ Procurement and Transplantation Network (OPTN)^15^.

According to the ABTO, the number of patients on the heart transplant waiting list has slightly increased from January to September 2019 and 2020 - absolute numbers were 260 and 277, respectively. These numbers do not meet those recorded in the USA, which presented a decrease by 34.2% in the number of patients on the waiting list for heart transplantation but an increase by 35.8% in the number of patients who died on the waiting list^16^.

### Lung

According to the ABTO, lung transplantation was mostly affected by the COVID-19 pandemic; it was only performed in Rio Grande do Sul and São Paulo states. RBT data indicated a decrease in the absolute number of lung transplants from January to September 2020, in comparison to the same period in 2019 - 72 and 39 transplants, respectively. Taking into consideration the evolution over the last 10 full years, it is possible to acknowledge an increase by 61 in the number of lung transplants performed in 2010, to a peak of 121 transplants, in 2018. Based on the projection calculated by ABTO for the last months of the year (Graphic 1), there would be 52 lung transplants performed in 2020, which would represent a decrease by 49% in comparison to the previous year - these numbers are against the upward trend - with some variations - in the last decade^9^. 

A study focused on evaluating transplants performed in the United States from January to April 2020 found a decrease by 40.04% in lung transplants, as well as an increase in the number of sick patients on the waiting list^16^. A six-week follow-up survey carried out with transplant surgeons and transplant ID physicians in the United States reported that 19% of lung transplant programs operated without restrictions in March 2020, against 29% in May of that same year. In addition, there was an increase from 4% to 24% in the number of programs that did not perform any lung transplant in the very same period^17^. A three-week French analysis indicated a decrease from 31 - in the number of elective and high emergency lung transplants in the same period of 2019 - to two, in 2020. In addition, there was a decrease in the number of lung donations due to the difficulty in implementing procedures to enable organ procurement by intensivists, who were under the heavy workload due to the COVID-19 pandemic^18^. The number of lung transplants in the UK as also decreased by 77% from March to May in 2019 and 2020, whereas the number of donors and patients who died on the waiting list increased^19^. Even in Australia, which recorded a significantly lower incidence of COVID-19, there was a decrease by 12% in the number of lung transplants20. Germany was the only country where the number of patients on the waiting list for lung transplant, the overall number of organ donors, as well as the mortality rate on the lung transplant waiting list did not have any significant changes in the first five months of 2020^21^. 

The number of patients on the waiting list for lung transplantation has slightly decreased from 199 (in 2019) to 195 (in 2020). A study conducted in Italy did not find significant changes on the waiting list due to the urgent nature of lung transplantation procedures, which cannot be postponed^22^. However, the number of new registrations on the waiting list in the United States and Germany indicated evident decrease in early 2020^16^ ^,^ ^21^. Picard et al.^18^ have emphasized that the higher risk of death (>50%) within one year is the main criterion to include individuals in the lung transplant waiting list, in France. Therefore, postponing the operation may lead to deaths that would be classified as indirectly caused by the COVID-19 pandemic.

### Liver

According to the ABTO, liver transplantation was the procedure least affected by the COVID-19 pandemic, since it decrease by only 10.8% in the three quarters of 2020, in comparison to the same period in 2019 - it increased by 16.9% in the 1^st^ quarter, by 29.1% in the 2^nd^ quarter, and by 11.9% in the 3^rd^ quarter. The highest decrease in the number of liver transplantations was recorded in the Northeastern region (37.1%), which was followed by the Southern region (21.1%), whereas the Southeastern (decrease by 0.3%) and Mid-Western regions (increase by 3.7%) have already recovered. Liver transplantations from living and deceased donors has decreased by 11%. The Federal District alone performed more than 20 transplants per million population (pmp)^9^. Similarly to Brazil, data from France and the USA also showed a reduced number of liver transplantations during the COVID-19 pandemic, in 2020^14^ ^,^ ^23^ ^,^ ^24^. 

France recorded a decrease by 22% in the number of liver transplants during the COVID-19 pandemic, as well as a sharp decrease in it during the lockdown periods. Such decrease can be explained by three reasons: most intensive care unit (ICU) beds are dedicated to critically ill patients infected with COVID-19; teams accounting for transplant operations were reassigned to take care of patients with respiratory symptoms, which hindered the logistics to harvest and transplant organs; and finally, some transplantation programs were suspended in France during the pandemic period^14^. The National Transplant Agency (ABM) and the Public Health Council (HCSP) started to recommend the screening of all donors in order to analyze the risk-benefit ratio to approve transplants. Consequently, the applied analysis to data in the second half of March 2019 showed approximately 60 liver transplants, which decreased to approximately 20 transplants in the same period in 2020^23^. 

The SARS-CoV-2 pandemic has led to an imbalance in the organ donation and procurement process in the USA, which resulted in decreased transplantation rates. According to data from the United Network for Organ Sharing (UNOS), there was a decrease by 25% in the number of livers from deceased donors between February and April 2020, whereas liver transplants from living donors recorded a low rate (5%), in March and April^24^.

The waiting list for liver transplantation in Brazil recorded a slight decrease from 1,124 patients in 2019, to 1,100 patients in 2020^9^. An Italian study has followed the same line, according to which a moderate decrease were observed during the pandemic in the transplant list. According to the authors of the aforementioned study, this happened due to the urgent nature of liver transplantation, which cannot be postponed^7^. A decrease in the number of patients on the waiting list was more significant in the USA; it reached 10.2%. However, the total number of deaths on the waiting list has increased by 7.7%, in comparison to the same period in 2019^16^.

### Pancreas

Based on data reported by the ABTO, the number of pancreatic transplant operations performed from January to September 2020 decreased in comparison to the number of procedures performed in the same period in 2019 - 108 and 125 transplants, respectively. Santa Catarina State recorded the highest decrease in the number of pancreatic transplants - from 11 to three transplants. São Paulo and Minas Gerais have kept the absolute number of transplants. Based on a 10-year period, the lowest transplant rate in these states was recorded in 2017 (only 113 transplants). On the other hand, there was a substantial increase in 2018 (146 transplants) and 2019 (175 transplants), which decreased again in 2020 (144 transplants)^9^. Difficulties faced by the transplant sector are often observed worldwide, as. countries such as the United States, France, the United Kingdom and Australia have reported a significant decrease in the number of transplant procedures^20^. 

The Netherlands recorded a decrease by 67% in its services during the pandemic period - the total number of pancreatic transplants dropped to zero^11^. On the other hand, Australia recorded an increased number of pancreatic transplants, despite the decline of the transplant services^20^. An American study about the practices and policies of transplant centers has found that 16% of pancreatic transplants were suspended, in comparison to 39% recorded in March of 2020^17^. According to the RBT, there was a decrease by 18% in pancreatic transplants in Brazil, in 2020. However, it is worth emphasizing that there was a decrease by 59.6% in this type of operations in the second quarter of that same year, as well as an increase by 23.1% in last semester - São Paulo (1.5 pmp) was the only state exceeding one transplant pmp^9^. 

### Kidney

According to data provided by the ABTO, when comparing 2019 to the same period in 2020, the number of kidney transplantations increased by 6.5% in the 1^st^ quarter, decreased by 43.2% in the 2^nd^ quarter and by 37.8% in the 3^rd^ quarter. Interestingly, there was a higher decrease in the number of kidney transplants in the 3^rd^ quarter in some regions due to late pandemic peak in the Southern region (54%), which was followed by the Northeastern (42%), Southeastern (30%) and Mid-Western (23%) regions^9^. 

The total number of kidney transplants significantly decreased from January to September 2020, in comparison to the same period in 2019 - 3,486 and 4,617 transplanted kidneys, respectively. The decrease in kidney transplant rates within these nine months reached 26.2% in 2019; 18.4% of it corresponded to deceased donors and 63.7% to living donors^9^. Likewise, according to data reported in Italy, France and in the United States, the COVID-19 pandemic led to a significant decrease in the number of kidney transplants^25^ ^,^ ^26^. The number of kidney transplants from deceased and living donors performed between March 15 and April 30, 2020, in the USA was, respectively, 24% and 87% smaller than expected, based on pre-epidemic data^27^. However, Austria was the only country to report that the total number of kidney transplants performed between January and June 2020 did not significantly differ from that of previous years (in comparison to the first six months of previous years). This outcome in Austria can be explained by restrictions implemented by the government at early pandemic stages, such as tests and home strategies^25^. 

Based on a survey conducted with 204 transplant institutions around the world, from May to June 2020, 75% of institutions had fully stopped performing kidney transplants from living donors due to governmental or managing restrictions. The assessment of living donors has also significantly decreased due to the COVD-19 pandemic, based on concerns about the safety of living donors associated with the risk of getting infected with the virus. In addition, 59% of the institutions reported to have temporarily stopped the assessment, which led to the drop in the total number of kidney transplants^28^ ^,^ ^29^. 

The ABTO annual projection for 2020 had anticipated 4,646 kidney transplants and showed sharp drop in the number of transplant recipients in Brazil, despite the trend to an increased number of kidney transplants and a total number of solid organ transplants in the last 10 years. Kidney transplantation reached its maximum peak in 2019, with a total number of 6,295 transplants^9^. Likewise, the USA registered the seventh year of records in the number of transplants, which, according to data released by the United Network for Organ Sharing (UNOS), recorded the total number of 39,719 transplants from January 2019 to December 2019 - it corresponded to an increase by 8.7% in comparison to 2018, which recorded 36,269 solid organ transplants. The temporary suspension of the living donor program in March 2020 was a significant setback, since donations decreased by almost 50%^30^. 

The number of patients on the waiting list for kidney transplant, according to the ABTO, increased in 2020, in comparison to the period from January to September 2019 - the absolute numbers were 25,724 and 23,630, respectively^9^. Likewise, a study conducted in the UK indicated that on March 5, 2020, there were 6,317 active patients on the waiting list for kidney transplant alone, in comparison to 4,649 in 2019 - this outcome corresponds to an increase from 16.3% to 35.2%. If the number of kidney transplants had not decreased, the list would have been reduced by 17 patients per month; however, the relative predicted model increase by up to 283 patients per month, which would result in the addition of 1,324 dialysis patients^31^. However, Austria was the only country to report that the number of patients on the waiting list did not increase from 30 June 2019 (n=627) to 2020 (n=595)^25^. On the other hand, the USA recorded a decrease by 18% in the number of new registrations on the waiting list for deceased-donor kidney transplant, which was likely due to delays in the evaluation of kidney transplant candidates^27^. 

The mean time spent on the waiting list for kidney transplant in the USA is three to five years and depends on several factors; patients who remain on the waiting list require kidney replacement therapy. Morbidity and mortality rates in dialysis patients are significantly higher than rates recorded for the non-dialysis population. In April 2020, the death rate of patients on the waiting list for kidney transplant in the USA increased by 43.0%, based on UNOS data^30^. 

## CONCLUSION

Organ donation campaigns should be carried out to make the population aware of the importance of maintaining organ donation, as well as to educate those patients on the list waiting for organs. In addition, strategies must be developed to keep the necessary infrastructure for active organ transplantation processess, as well as to mitigate the impacts of the COVID-19 pandemic on these patients.
